# Long Non-Coding RNA HOXA10-AS Promotes the Migration and Invasion of Glioblastoma Cells by Serving as a Competing Endogenous RNA for miR-99a-3p to Upregulate ITGB5 Expression

**DOI:** 10.32604/or.2025.068313

**Published:** 2025-11-27

**Authors:** Yingjie Wang, Wanlin Dong, Can Wang, Zirui Li, Yongqiang Wang, Qi Li, Cheng-Ya Dong

**Affiliations:** 1 Department of Neurology, China National Clinical Research Center for Neurological Diseases, Beijing Tiantan Hospital, Capital Medical University, Beijing, 100071, China; 2 Department of Neuro-oncology, Cancer Center, Beijing Tiantan Hospital, Capital Medical University, Beijing, 100071, China

**Keywords:** HOXA10-AS, competing endogenous RNA, migration, bioinformatics analysis, glioblastoma

## Abstract

**Objectives:**

Glioblastoma is a prevalent malignant brain tumor, and the actions of the long non-coding RNA HOXA10-AS in its invasion and migration remain unclear. Here, the function of HOXA10-AS in glioblastoma cell invasion and migration and associated mechanisms were investigated.

**Methods:**

HOXA10-AS was knocked down in glioblastoma cells, and Transwell and wound healing assays were conducted to elucidate its impacts on cell invasion and migration. Western blotting and quantitative reverse transcription polymerase chain reaction (qRT-PCR) assessed HOXA10-AS’s impact on the epithelial-mesenchymal transition (EMT). Microarray analysis identified differentially expressed genes, complemented by bioinformatics approaches to explore potential molecular participants and pathways. Rescue experiments validated our findings.

**Results:**

HOXA10-AS knockdown significantly inhibits glioblastoma cell migration, invasion, and the EMT process. Specifically, HOXA10-AS siRNA transfection significantly reduced the migratory capacity of A172 cells by 50.5% and U251 cells by 61.4%, as well as their invasive capacities by 33.8% and 58.5%, respectively (all *p* < 0.05). HOXA10-AS acts as an miR-99a-3p sponge, and pathway analysis identified processes linked to tumorigenesis and metastasis, along with nine hub genes. HOXA10-AS upregulates the expression of integrin subunit beta 5 (ITGB5) through a competing endogenous RNA mechanism. The reduced tumorigenic behavior of glioblastoma cells due to HOXA10-AS knockdown can be rescued by ITGB5 overexpression or miR-99a-3p inhibitor.

**Conclusion:**

These results indicate that HOXA10-AS promotes tumorigenic behavior in glioblastoma cells by regulating the EMT-like process and functioning as an miR-99a-3p sponge to modulate ITGB5 levels, providing insights into glioblastoma development and potential therapeutic targets.

## 1 Introduction

Glioblastoma is a primary malignancy of the central nervous system, originating from glial cells. These tumors are characterized by an increased prevalence and poor prognosis [1–4]. The invasion and migration of glioblastoma cells are significant contributors to treatment failure and unfavorable outcomes. Glioblastoma is currently treated with surgery, radiotherapy, and systemic therapy (chemotherapy, targeted therapy) [5–7]. Unfortunately, the vast majority of patients will experience an early tumor progression or recurrence despite the application of aggressive multimodality treatment approaches. Moreover, glioblastomas are particularly challenging, with < 5% survival rates for 5 years [8,9]. A deeper understanding of the mechanisms underlying glioblastoma cell invasion and migration is crucial for developing new therapeutic strategies and exploring potential therapeutic targets for glioblastoma treatment [10].

The competitive endogenous RNA (ceRNA) hypothesis posits that non-coding RNAs, like circular RNAs (circRNAs) and long non-coding RNAs (lncRNAs), control gene levels via competitive interactions with microRNAs (miRNAs) [11–13]. Among these, lncRNAs are > 200 nucleotides long, ubiquitous RNA molecules [14–16]. Studies have indicated that they are linked with the incidence and development of various malignancies and modulate the differentiation, migration, proliferation, and invasion of tumor cells [17,18]. In the context of glioblastoma, lncRNAs have demonstrated multiple functions, including early tumor detection, prognosis prediction in patients with tumors, and modulation of the infiltration of immune cells [19–21]. Many studies have highlighted the potential therapeutic applications of lncRNAs in glioblastoma [22–24]. Yet many areas remain to be explored. Our earlier study described the identification of a novel lncRNA, designated HOXA10-AS, from the antisense strand of the gene encoding homeobox A10 (*HOXA10*), and demonstrated its significant upregulation in glioblastoma, influencing cancer cell proliferation [25]. Subsequently, an investigation of non-coding transcripts in pan-cancer confirmed the prognostic roles of the onco-lncRNA HOXA10-AS in glioblastomas, revealing that its upregulation was associated with poor prognosis in IDH-mutant (MUT) glioblastoma and isocitrate dehydrogenase (IDH)-wild-type (WT) patients [26]. In certain cancer types, like esophageal carcinoma and oral cancer, HOXA10-AS can induce cancer cell proliferation and metastasis by either stabilizing its neighboring gene HOXA10 [27] or serving as a modular scaffold for the processing of TP63 mRNA [28]. Additionally, HOXA10-AS can elevate migration, proliferation, and invasion in gastric cancer by elevating HOXA10 levels via the p38/MAPK/STAT3 axis [29]. Furthermore, in pancreatic cancer metastasis, HOXA10-AS has been verified to contribute to tumor aggressiveness by competing with HTR1D and miR-340-3p as a ceRNA [30]. However, despite the critical role of glioblastoma cell invasion and migration in treatment failure and poor outcomes, there is a paucity of studies investigating whether HOXA10-AS influences these processes and elucidating its distinct role and responsible molecular pathways.

This research aimed to elucidate the modulatory functions and potential pathways of HOXA10-AS in glioblastoma. Specifically, we knocked down the HOXA10-AS in glioblastoma cells and conducted Transwell and wound-healing assays to examine its effects on invasion and migration. The differentially expressed genes (DEGs) after HOXA10-AS knockdown were determined via Microarray analysis. In addition, the bioinformatics assay was carried out to explore the potential molecular players and signal pathways associated with this process. Finally, the study conducted experiments to validate the findings from the bioinformatic analysis.

## 2 Materials and Methods

### 2.1 Cells

The human glioblastoma cell lines A172 and U251 were acquired from the ATCC (Manassas, VA, USA), with authentication using short tandem repeat (STR) profiling. The cells were propagated in 5% penicillin-streptomycin and 10% fetal bovine serum (FBS; Thermo Fisher Scientific, Waltham, MA, USA) in DMEM (Thermo Fisher Scientific) at 37°C with 5% CO_2_. When between 80% and 90% confluent, cells were subcultured using 0.25% trypsin. Furthermore, mycoplasma contamination status was regularly monitored and confirmed to be negative for both cell lines throughout the study.

### 2.2 HOXA10-AS Knockdown

Lipofectamine RNAiMAX Transfection Reagent (Invitrogen, Thermo Fisher Scientific) was employed for transfection of the HOXA10-AS siRNA constructs or control scrambled siRNAs into A172 and U251 cells, per the kit’s guide. The siRNA sequences, including the sequences of scrambled siRNA (negative control) and the two HOXA10-AS siRNA, were acquired from GenePharma (Shanghai, China) (Table 1).

**Table 1:** siRNA oligonucleotides

siRNA	Sequences (5^′^-3^′^)
si-HOXA10-AS#1	S: GACGAUUUCAACUGAAGUATT
	A: UACUUCAGUUGAAAUCGUCTT
si-HOXA10-AS#2	S: CACCAAGCAAACACAAAGATT
	A: UCUUUGUGUUUGCUUGGUGTT
si-NC	S: UUCUCCGAACGUGUCACGUTT
	A: ACGUGACACGUUCGGAGAATT

Note: S, sense; A, antisense.

### 2.3 RNA Isolation and Quantitative Reverse Transcription Polymerase Chain Reaction (qRT-PCR)

Whole A172 and U251 cellular RNA was harvested using TRIzol (Invitrogen), and the PrimeScript RT-PCR Kit (Takara Biotech, Dalian, China) was then employed to reverse-transcribe the acquired RNA into cDNA. PCR amplification was conducted with Universal SYBR Green Master Mix (Takara Biotech). Specific primers for HOXA10-AS, E-cadherin, INSR, EGFR, FGF12, ITGB5, Vimentin, SRC, VEGFC, N-cadherin, MRAS, PDPK1, PIP5K1C, and GAPDH are detailed in Table 2. The reference was GAPDH, and relative gene levels were evaluated using the comparative threshold cycle (Ct) method.

**Table 2:** qRT-PCR primer sequences

Gene name	Primer forward sequence (5^′^-3^′^)	Primer reverse sequence (5^′^-3^′^)
HOXA10-AS	CCCAGTAAGCCAAAGTCAAGCC	CTGAGGTCAATGGTGCAAAGG
E-cadherin	CGGACGATGATGTGAACACC	TTGCTGTTGTGCTTAACCCC
N-cadherin	ATATTTCCATCCTGCGCGTG	GTTTGGCCTGGCGTTCTTTA
Vimentin	GAGTCCACTGAGTACCGGAG	ACGAGCCATTTCCTCCTTCA
EGFR	AGGCACGAGTAACAAGCTCAC	ATGAGGACATAACCAGCCACC
FGF12	CCAGCAAGAATCAGGCCGAG	TCGGTACAAAATGTGATGAGGG
INSR	AAAACGAGGCCCGAAGATTTC	GAGCCCATAGACCCGGAAG
ITGB5	AACTCGCGGAGGAGATGAG	GGTGCCGTGTAGGAGAAAGG
SRC	GAGCGGCTCCAGATTGTCAA	CTGGGGATGTAGCCTGTCTGT
VEGFC	GAGGAGCAGTTACGGTCTGTG	TCCTTTCCTTAGCTGACACTTT
MRAS	TTCCTCATCGTCTACTCCGTC	AGGATCATCGGGAATGACTCC
PDPK1	GGAACAGCGCAGTACGTTTCT	CTCGTTTCCAGCTCGGAATGG
PIP5K1C	AGACCGTCATGCACAAGGAG	CAGTACAGCCCATAGAACTTGG
GAPDH	GCACCGTCAAGGCTGAGAAC	TGGTGAAGACGCCAGTGGA

### 2.4 Western Blot Analysis

Cells were harvested for protein or RNA extraction approximately 24 h post-transfection. Protein (40 μg), extracted from the A172 and U251 cells using a commercial kit (#BC3710, Solarbio, Beijing, China), was subjected to 10% or 12% SDS-PAGE, transferred to PVDF membranes (Millipore, Bedford, MA, USA), blocked for 2 h with 5% BSA or skim milk in TBST, and then treated at 4°C with primary antibodies against N-cadherin (D4R1H, #13116), Vimentin (D21H3, #5741), β-actin (13E5, #4970), and E-cadherin (24E10, #3195) (all 1:1000 dilution; CST, Danvers, MA, USA) overnight. The blots were then treated with an HRP-labeled secondary antibody (#7074; CST) for 1 h at ambient temperature. Chemiluminescence was assessed using a G:BOX imaging system (G:BOX Chemi XX9, Syngene, Cambridge, UK). Quantifications of immunoblots were performed using ImageJ (version 1.48v; National Institutes of Health, Bethesda, MD, USA), and the densitometry data were normalized to the loading control.

### 2.5 Wound Healing Assays

Scratch tests were conducted to assess cell migration rates. Briefly, after transfection, both cell lines (1 × 10^5^ cells/6-well plate) were seeded. Once the monolayer formed, cells were scratched using a pipette tip, after which the cells were rinsed twice with 0.1M PBS (pH 7.4) to remove debris and propagated in 1% FBS-containing media for 24 h. Wound healing assays were initiated 24 h post-transfection to ensure optimal siRNA-mediated knockdown efficiency, and wound closure was monitored for 24 h (total experimental duration: 48 h post-transfection). After 0 and 24 h, the wound closure area was analyzed at five randomly selected fields. The wound healing rate was calculated via ImageJ software.

### 2.6 Transwell Migration Assay

A172 and U251 cells (5 × 10^4^) were grown in the top compartments of Transwell inserts (Corning, Corning, NY, USA) containing serum-free DMEM in 5% CO_2_ at 37°C. The lower chambers contained DMEM + 10% FBS. After 24 h, cells on the lower surface were preserved for 30 min with 4% paraformaldehyde and then stained for 20 min using 0.1% crystal violet. After removal of non-migrated cells, the migrated cells in five random fields per membrane were enumerated under 200× magnification and imaged under inverted microscopy (Zeiss Axio Vert. A1, Carl Zeiss AG, Oberkochen, Germany). The average number of cells per field for the control group (si-NC) typically ranged between 143 and 167 in A172 cells and 152 and 218 in U251 cells across replicates. Data are given as the mean number of cells per field or as a relative percentage of the si-NC control group (set to 100%).

### 2.7 Invasion Assays

Upper Transwell compartments were precoated with Matrigel (BD Biosciences, Franklin Lakes, NJ, USA), and A172 and U251 cells in serum-free DMEM were added. The lower compartments were filled with medium (600 μL). After 24 h, cells were rinsed, and cell suspensions were introduced to the insert chambers for 24 h. The chambers were then removed, rinsed with 0.1M PBS (pH 7.4), and the reverse side of the membrane was preserved and stained as described above. Cells were counted and imaged as above. The average number of cells per field for the control group (si-NC) typically ranged between 122 and 158 in A172 cells and between 445 and 502 in U251 cells across replicates.

### 2.8 Microarray

Total A172 cell RNA was isolated using TRIzol (Invitrogen) and purified with an RNeasy Mini Kit (#74104; Qiagen, Hilden, Germany). Biotinylated cDNA was synthesized using the Ambion^®^ WT Expression Kit (#4411974; Life Technologies, Bleiswijk, The Netherlands), as directed, using 250 ng of total RNA. After labeling, hybridization of fragmented cDNA was carried out for 16 h at 45°C on the Clariom™ D Human Assay (#902922; Affymetrix, Santa Clara, CA, USA). Then, the Affymetrix Fluidics Station 450 was stained, followed by array scanning with the help of the Affymetrix^®^ GeneChip Command Console on the GeneChip^®^ Scanner 3000 7 G. The Transcriptome Analysis Console (version: 4.0.1) with the Robust Multichip Analysis (RMA) algorithm was employed with Affymetrix default settings and global scaling to normalize raw data (.cel files). Values were presented as log_2_ RMA signal intensity. The *limma* R package (version 3.36.5) was employed to identify DEGs. Benjamini-Hochberg correction was applied for multiple testing, with thresholds set for significant up-regulation and down-regulation: FDR < 0.05, |fold change| > 2.0, and *p*-value < 0.05.

### 2.9 KEGG and Bioinformatics Analyses

The pathways associated with the DEGs were investigated using KEGG enrichment, with enrichment scored as −log_10_ (*p*-value) to assess pathway significance and specificity. Additionally, the miRNAs with the highest predicted binding affinities were identified using miRanda software (version: 3.3a), which evaluates both thermodynamic stability scores and sequence conservation scores. Hierarchical clustering analysis was performed on the 37 significantly downregulated miR-99a-3p target mRNAs linked with cellular invasion and migration. The resulting clusters were visualized as a heatmap coupled with dendrograms using GraphPad Prism 8.0. The analysis was based on the log_2_-transformed fold-change values (HOXA10-AS knockdown vs. control) of these mRNAs.

### 2.10 Plasmid Construction and Transfection

The pmirGLO luciferase reporter vector, containing both firefly and Renilla luciferase genes, was used to clone the miR-99a-3p binding sequence and its mutated form from the HOXA10-AS 3^′^UTR, as well as the ITGB5 3^′^UTR sequences (GenePharma). Additionally, we constructed a plasmid based on the pCDH backbone to facilitate the overexpression of the HOXA10-AS 3^′^UTR. The human ITGB5 overexpression plasmid was graciously provided by Mailgene (Mailgene plasmid: MH02016; http://www.mailgene.cn/index/index/plasmiddetail/id/149.html (accessed on 01 January 2025); RRID: Mailgene_MH02016). For transfection, A172 cells (1 × 10^5^ cells/6-well plates) were seeded for 24 h until they reached a density of 60%–70%. Transfection of plasmids and miRNA mimics or inhibitors (GenePharma) was carried out using Lipofectamine 2000 reagent (Invitrogen), per the kit’s protocol.

### 2.11 Luciferase Reporter Assays

HEK-293 T cells were purchased from the ATCC and authenticated as above. Mycoplasma contamination status was regularly monitored and confirmed to be negative throughout the study. HEK-293 T cells (1 × 10^5^) were grown in 24-well plates and co-transfected with the reporter vector and miR-99a-3p mimics. Assays were performed using a Dual Luciferase Reporter Assay Kit (#E1910; Promega, Madison, WI, USA). Cell lysis and luciferase detection were carried out 24 h post-transfection. *Renilla* activity was employed as a standard to normalize Firefly luciferase activity, and the data were depicted as a percentage of luciferase activity compared to control cells.

### 2.12 Glioblastoma Data Download and Differential Expression Analysis

Two miRNA microarray datasets related to miR-99a-3p expression were imported from Gene Expression Omnibus (GEO) datasets. The GSE42657 dataset comprised 33 fresh-frozen pediatric glioblastoma samples and 9 healthy brain tissue samples, while the GSE61710 dataset comprised 7 adult glioblastoma samples and 5 healthy tissue samples. GEO2R was utilized to analyze miR-99a-3p levels in glioblastoma tissue relative to normal brain samples across both datasets.

The mRNA expression profile of the ITGB5 gene from The Cancer Genome Atlas (TCGA) was analyzed using the GlioVis website (http://gliovis.bioinfo.cnio.es/, accessed on 01 January 2025) [31] on 18 July 2024. This analysis included 226 cases of grade II glioblastomas, 244 cases of grade III glioblastomas, and 150 cases of grade IV glioblastomas. We searched for ITGB5, generated visualizations using the GlioVis platform (Version 0.20) to compare its expression across different glioblastoma grades, and performed statistical analyses to elucidate the significance of the observed differential expression.

### 2.13 Survival Analysis of ITGB5 in Glioblastomas

The survival outcomes of high and low ITGB5 expression glioblastoma patients were evaluated using TCGA (n = 160) and China Glioblastoma Genome Atlas (CGGA, n = 84, https://www.cgga.org.cn/about.jsp, accessed on 01 January 2025) databases. Kaplan-Meier survival curves were generated for each dataset to illustrate the survival probability over time between the high- and low-expression groups.

### 2.14 Statistical Assessment

All data are indicated as mean ± standard error of the mean (SEM). To confirm the Gaussian distribution of the values, the Shapiro–Wilk normality test was carried out. All statistical measurements were carried out using GraphPad Prism 8.0. Intergroup comparisons were assessed using unpaired two-tailed *t*-tests, whereas for multiple-group comparisons, one-way ANOVA followed by Dunnett’s test was carried out. All assays were performed in triplicate at a minimum. The *p*-value of < 0.05 depicts statistical significance.

## 3 Results

### 3.1 HOXA10-AS Knockdown Suppresses the Invasion and Migration of Glioblastoma Cells

**Figure 1:**
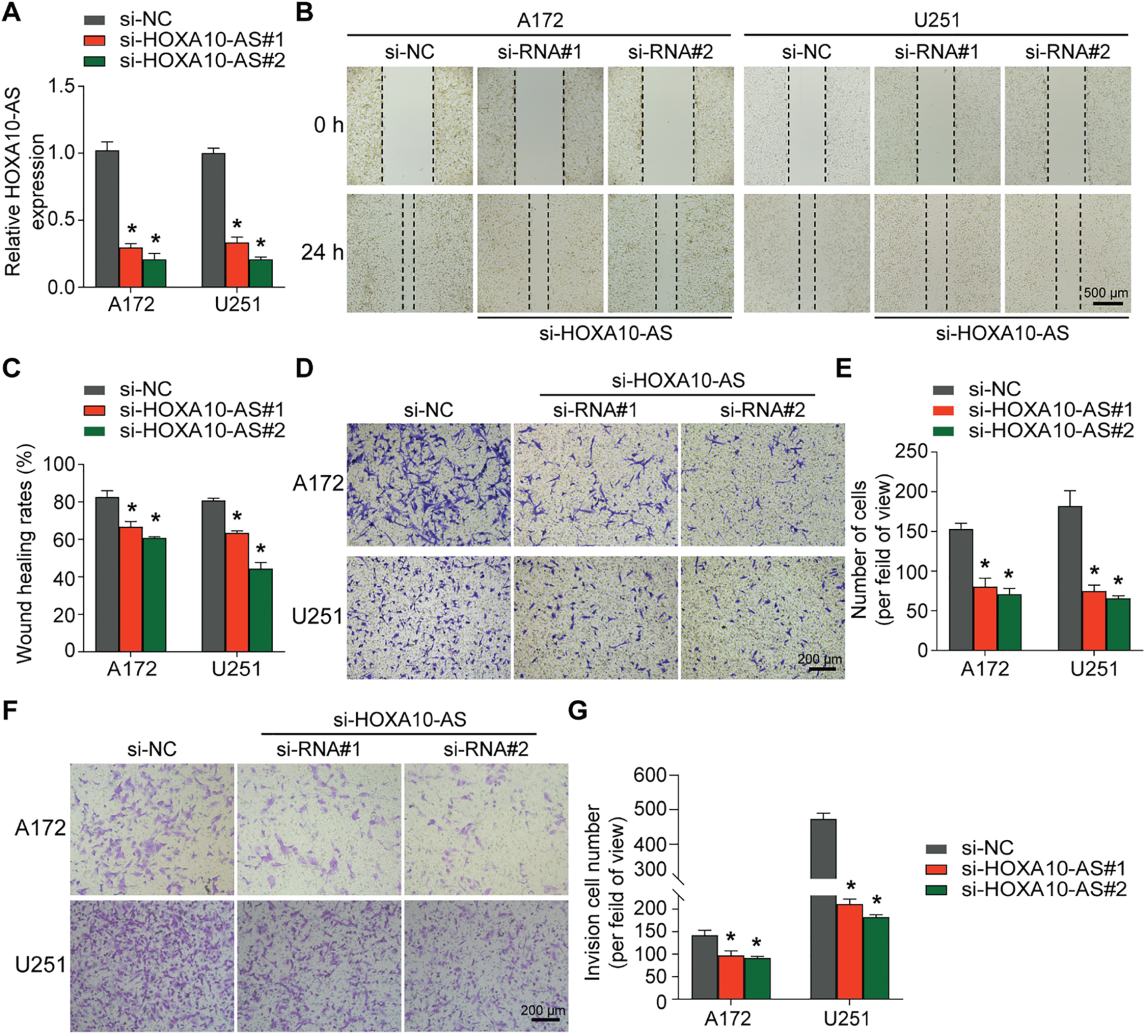
HOXA10-AS knockdown inhibits glioblastoma invasion and migration. **(A)** U251 and A172 cells were transfected with HOXA10-AS siRNA#1, HOXA10-AS siRNA#2, or control siRNA, and the knockdown efficiency of HOXA10-AS was assessed using qRT-PCR 24 h after transfection. **(B)** The migration capability of 48 h post-transfected A172 and U251 cells was evaluated via the wound healing assay. Scale bar: 500 μm. **(C)** Quantification of the wound healing results. **(D)** The migration capability of 48 h post-transfected A172 and U251 cells was evaluated via Transwell assays. Scale bar: 200 μm. **(E)** Quantification of migration results. **(F)** An invasion assay was carried out 48 h after transfection to elucidate A172 and U251 cells’ invasive potential. Scale bar: 200 μm. **(G)** Quantification of invasion results. Values are shown as mean ± SEM of 3 independent assays. *p < 0.05 relative to the si-NC group

In our previous study, we assessed HOXA10-AS levels in A172 and U251 glioblastoma cell lines and revealed that HOXA10-AS was substantially increased in both cell lines (approximately 6000-fold in A172 and 2500-fold in U251 compared to normal human astrocytes) [25]. Consequently, we selected A172 and U251 for further evaluation of the impact of HOXA10-AS on invasion and migration in glioblastoma. HOXA10-AS siRNA was transfected into both A172 and U251 cells, which resulted in a significant downregulation of HOXA10-AS following knockdown (Fig. 1A). Subsequently, Transwell and wound-healing assays were performed to examine changes in A172 and U251 cells’ invasion and migration capabilities after HOXA10-AS knockdown. Wound healing analysis revealed that the migration of both cell lines transfected with si-HOXA10-AS was significantly reduced: A172 displayed a 50.5% reduction in transwell migration, while U251 achieved a 61.4% reduction compared to cells transfected with control scramble interference RNA (*p* < 0.05, Fig. 1B,C). Additionally, the Transwell assays demonstrated markedly reduced migratory capacity in both A172 and U251 cells with HOXA10-AS knockdown, showing reductions of 33.8% and 58.5% respectively, relative to the control group (*p* < 0.05, Fig. 1D,E). In addition, Transwell assays incorporating Matrigel indicated that the invasive ability of these cells was markedly inhibited after HOXA10-AS silencing relative to cells treated with control interference RNA (Fig. 1F,G). These findings indicate that HOXA10-AS is crucially associated with the invasion and migration properties of glioblastoma cells.

### 3.2 HOXA10-AS Knockdown Inhibits the EMT-Like Process in Glioblastoma Cells

**Figure 2:**
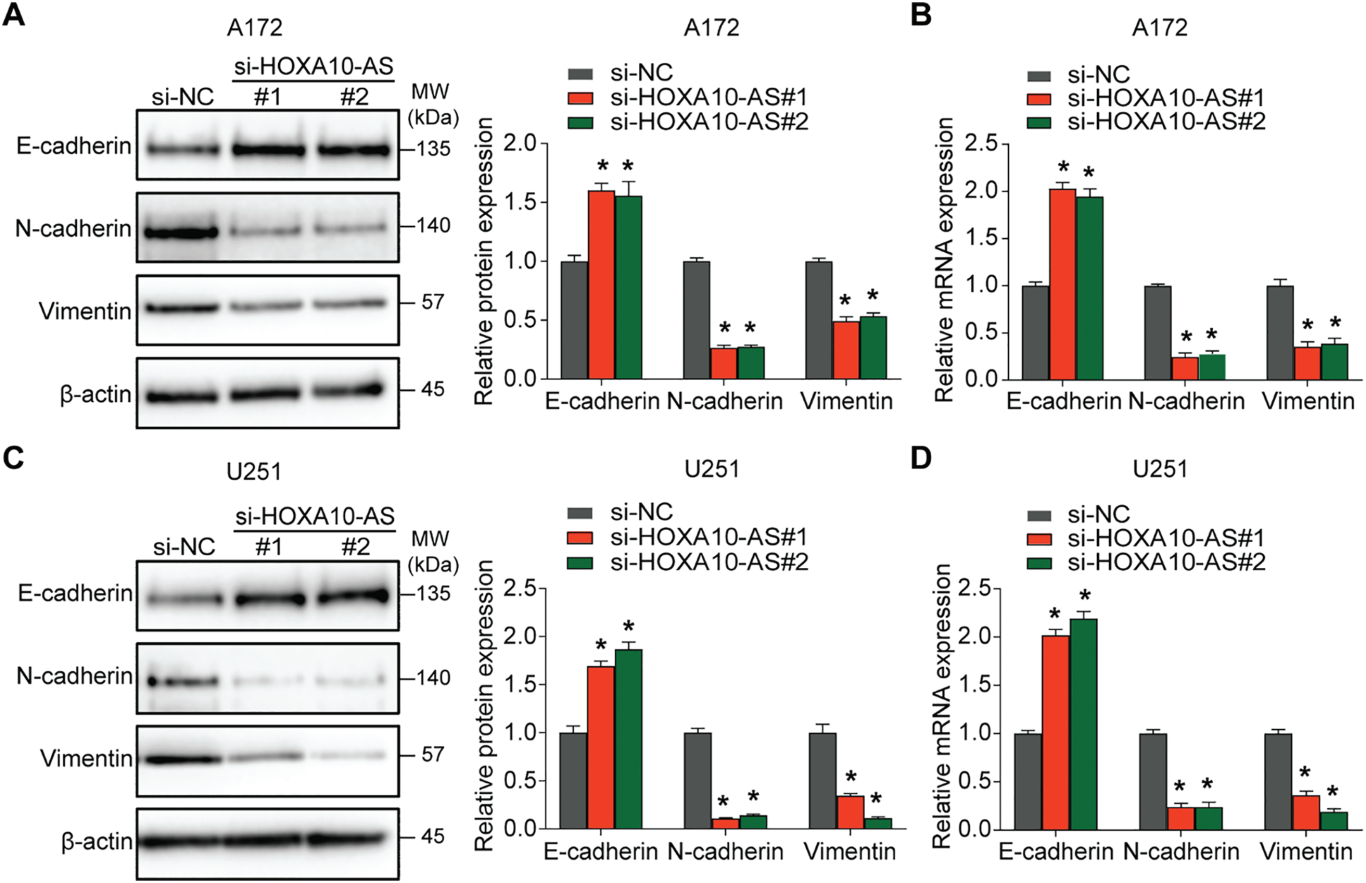
HOXA10-AS knockdown inhibits the EMT in glioblastoma. **(A)** Western blotting was carried out to elucidate E-cadherin, Vimentin, and N-cadherin expression in HOXA10-AS-silenced A172 cells. The corresponding protein levels are quantified in the adjacent graph. **(B)** qRT-PCR was utilized to measure the mRNA levels of EMT-related markers E-cadherin, Vimentin, and N-cadherin in HOXA10-AS siRNA or control siRNA-transfected A172 cells. **(C)** Western blotting was carried out to elucidate E-cadherin, Vimentin, and N-cadherin expression in HOXA10-AS-silenced U251, with quantifications displayed in the adjacent graph. **(D)** The mRNA levels of EMT markers E-cadherin, N-cadherin, and Vimentin in U251 cells transfected with either HOXA10-AS siRNA or control siRNA were determined using qRT-PCR. Values are shown as mean ± SEM of 3 independent assays. *p < 0.05 relative to the si-NC group

Given the importance of EMT in glioblastoma invasion and metastasis, this study also investigated the impact of HOXA10-AS on EMT. The results of Western blot and qRT-PCR analyses demonstrated that knockdown of HOXA10-AS in A172 cells significantly increased the expression of the epithelial marker E-cadherin, while markedly reducing the levels of the mesenchymal markers N-cadherin and Vimentin compared to controls (Fig. 2A,B). Similarly, in U251 cells, both Western blot and qRT-PCR analyses showed a significant elevation in E-cadherin levels, whereas N-cadherin and Vimentin levels declined after HOXA10-AS knockdown compared to controls (Fig. 2C,D). These results indicate that HOXA10-AS knockdown significantly alleviates the EMT-like processes in glioblastoma cells, suggesting a potential regulatory role in promoting their invasive and migratory characteristics.

### 3.3 HOXA10-AS Is an miR-99a-3p Sponge in Glioblastoma Cells

**Figure 3:**
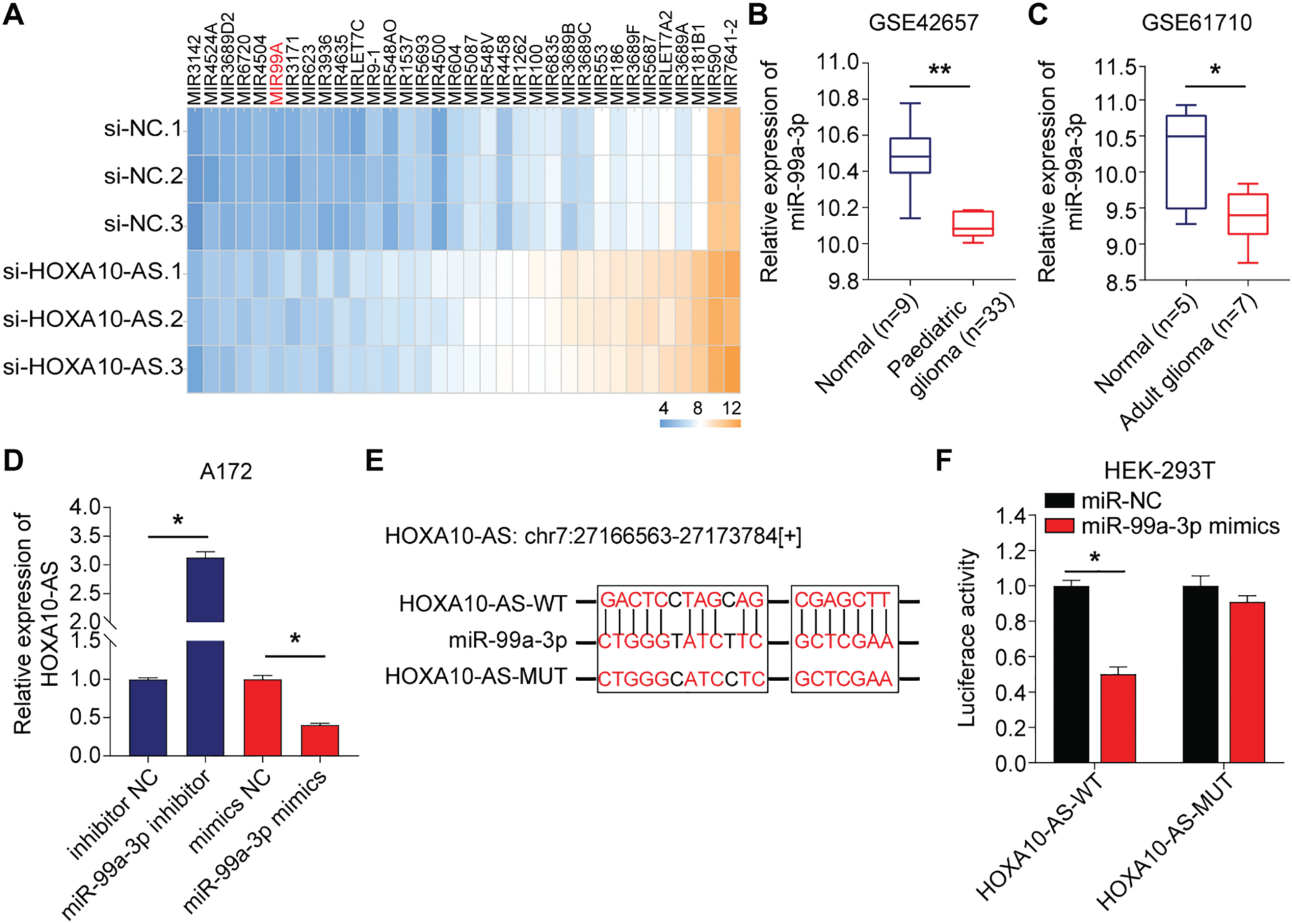
HOXA10-AS is an miR-99a-3p sponge in glioblastoma cells. **(A)** A heatmap displaying 34 miRNAs that are significantly upregulated in A172 cells transfected with HOXA10-AS siRNA (fold change > 2, adjusted *p* < 0.05) relative to control siRNA. **(B, C)** Analysis of differentially expressed miR-99a-3p in human glioblastomas based on GSE42657 (pediatric glioblastoma) and GSE61710 (adult glioblastoma) databases. **(D)** qRT-PCR assessment of HOXA10-AS levels in miR-99a-3p inhibitors or mimics transfected A172 cells. **(E)** The binding sequence of miR-99a-3p in the HOXA10-AS 3^′^UTR and its mutant form. **(F)** Luciferase activity in HEK-293 T cells co-transfected with luciferase reporter constructs comprising HOXA10-AS MUT or WT or sequences along with miR-99a-3p mimics or controls. Values are indicated as mean ± SEM of 3 independent assays. **p < 0.01, *p < 0.05

To investigate the regulatory mechanisms of HOXA10-AS in glioblastoma invasion and migration, we transfected A172 cells with siRNA targeting HOXA10-AS and analyzed the changes in microRNA (miRNA) levels. Relative to the control group, the A172 cells with HOXA10-AS knockdown exhibited a significant upregulation of 69 miRNA precursors. After deduplication, we identified 63 miRNA precursors that were substantially increased (*p* < 0.05, fold change > 2), of which 34 were formally recognized by the Human Gene Nomenclature Committee (HGNC) and are available on their official website (Fig. 3A). With the help of miRanda software, we predicted the target genes of these 34 upregulated miRNAs, and the findings showed that only the mature form of miR-99a-3p (the precursor of miR-99A) was predicted to target HOXA10-AS. The previous literature reports the association of miR-99a-3p with tumor invasion and metastasis [32]. We directed our investigation toward miR-99a-3p. Using the GEO2R tool, we analyzed miR-99a-3p levels in pediatric brain tumors (GSE42657) and adult glioblastomas (GSE61710) from the GEO database. It was found that the expression levels of miR-99a-3p were decreased in both pediatric and adult glioblastomas compared to their respective normal control groups (Fig. 3B,C).

The influence of miR-99a-3p modulation on HOXA10-AS levels was analyzed to investigate potential targeting interactions between miR-99a-3p and HOXA10-AS. Relative to the controls, the levels of HOXA10-AS were markedly enhanced after the miR-99a-3p inhibitor transfection, which was substantially reduced after miR-99a-3p mimics transfection, indicating that miR-99a-3p modulates HOXA10-AS (Fig. 3D). Next, the dual-luciferase assays were utilized to verify the direct interaction between miR-99a-3p and the HOXA10-AS 3^′^UTR. We designed HOXA10-AS WT and HOXA10-AS MUT firefly luciferase plasmids (Fig. 3E) to encompass all binding sites between the HOXA10-AS 3^′^UTR and miR-99a-3p, which were then co-transfected with miR-99a-3p or miR-NC mimics into HEK-293T cells. Furthermore, the luciferase activity in the HOXA10-AS-WT and miR-99a-3p mimic co-transfected groups was markedly reduced relative to the control group, while no marked changes were observed in the HOXA10-AS-MUT and miR-99a-3p mimic co-transfection group (Fig. 3F). These results showed that the HOXA10-AS gene is the target of miR-99a-3p.

### 3.4 Screening of Migration/Invasion-Related Differentially Expressed mRNA Regulated by miR-99a-3p after HOXA10-AS Knockdown

**Figure 4:**
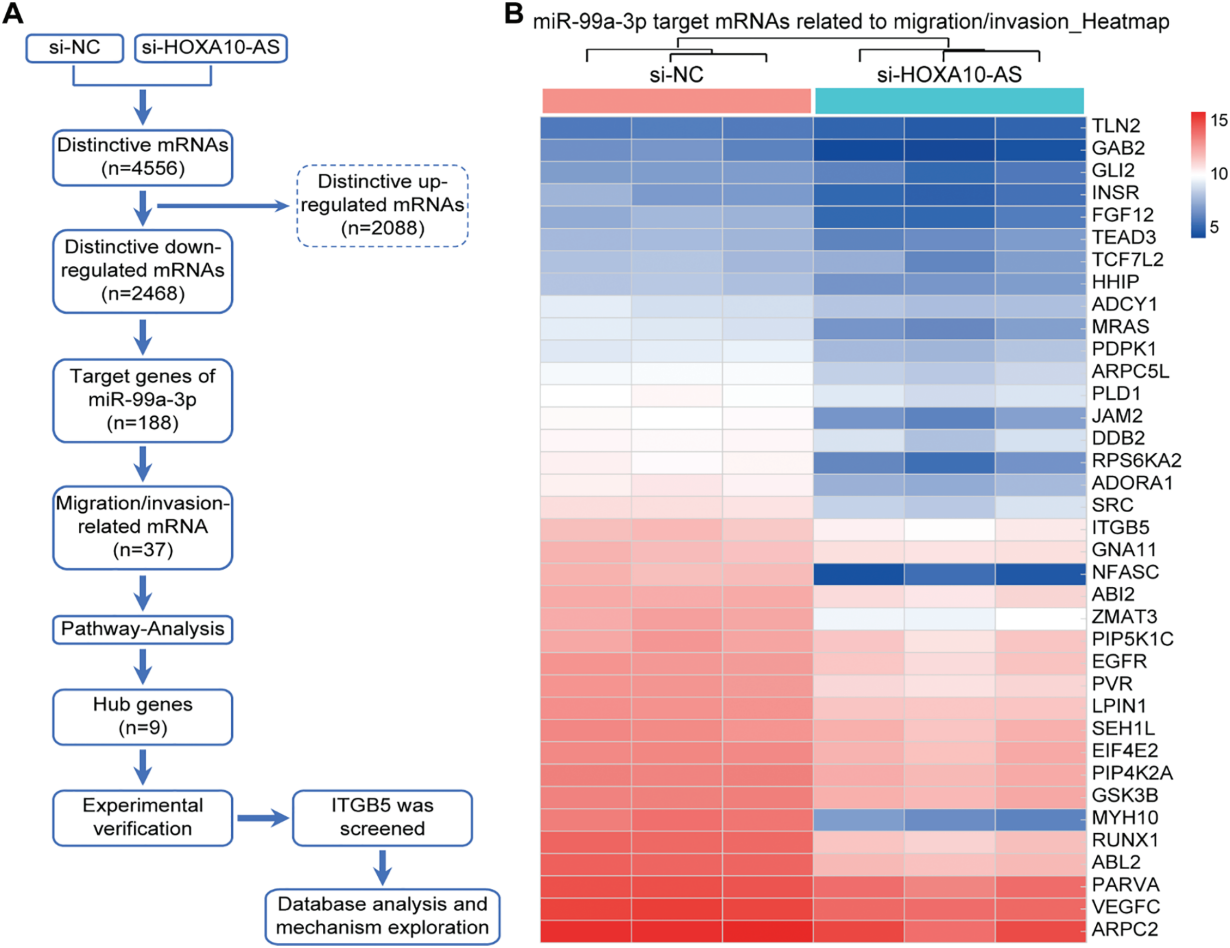
Screening of differentially expressed mRNAs associated with migration and invasion that are regulated by miR-99a-3p following HOXA10-AS knockdown. **(A)** Schematic diagram of the workflow used to identify mRNAs down-regulated by miR-99a-3p that are related to migration and invasion in A172 cells after HOXA10-AS knockdown. **(B)** Clustering analysis of 37 mRNAs related to cellular invasion and migration in A172 cells with HOXA10-AS knockdown vs. controls (*p* < 0.05, fold change > 2). Red represents high levels, and blue represents low levels

**Figure 5:**
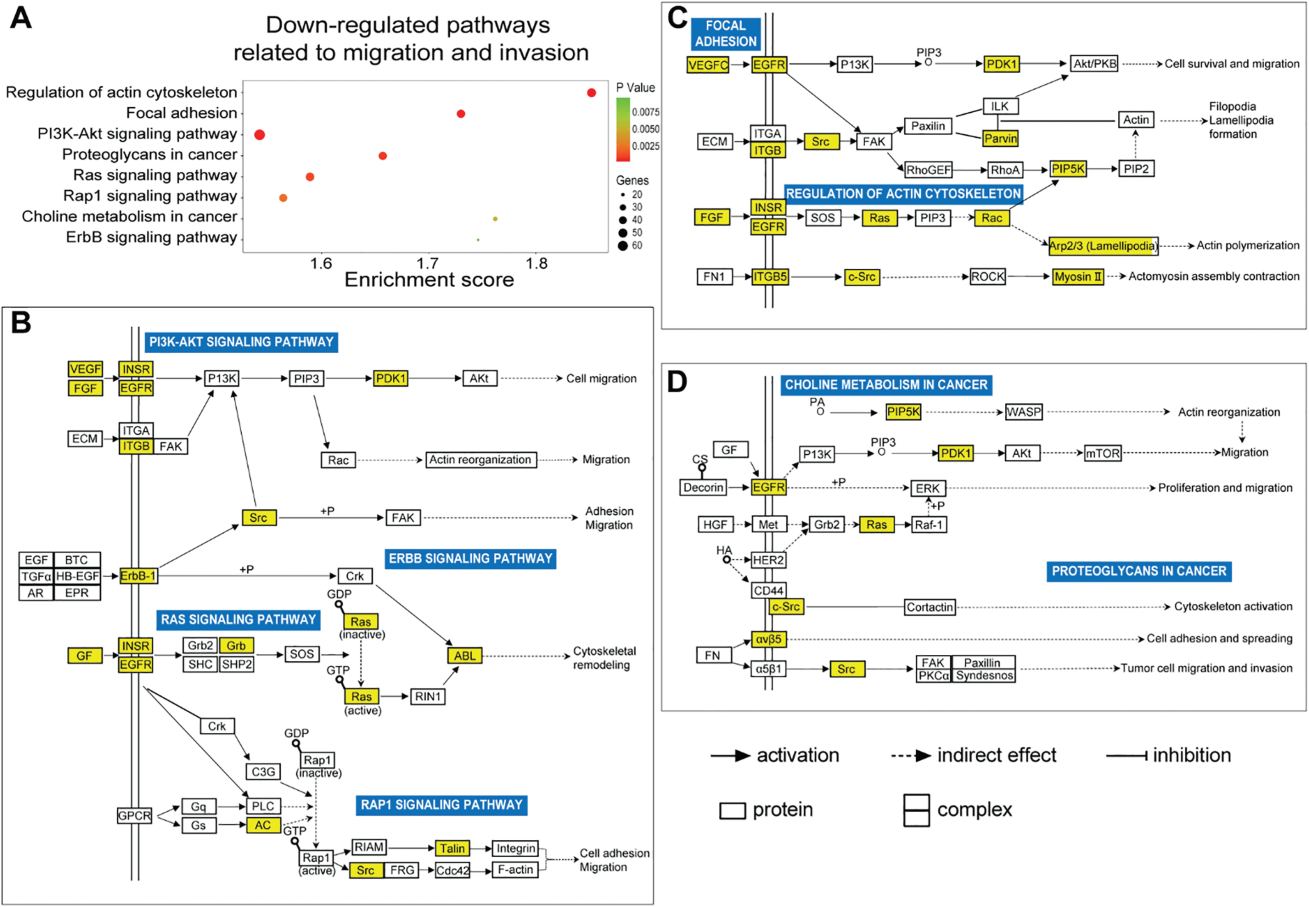
Prediction of biological functions of downregulated mRNAs based on KEGG pathway analysis. **(A)** KEGG analysis showed a scatter plot of downregulated signaling pathways associated with migration and invasion (*p* < 0.05). **(B–D)** Schematic diagram of signaling pathways associated with 37 downregulated migration- and invasion-related target mRNAs of miR-99a-3p, whose expression significantly decreased due to HOXA10-AS knockdown in glioblastoma cells. Yellow squares indicate genes affected by HOXA10-AS knockdown within the relevant pathways, while white squares represent other genes in the pathway that are not regulated by HOXA10-AS knockdown. Solid and dashed arrows denote direct and indirect correlations, respectively. The symbol (+p) indicates phosphorylation

**Figure 6:**
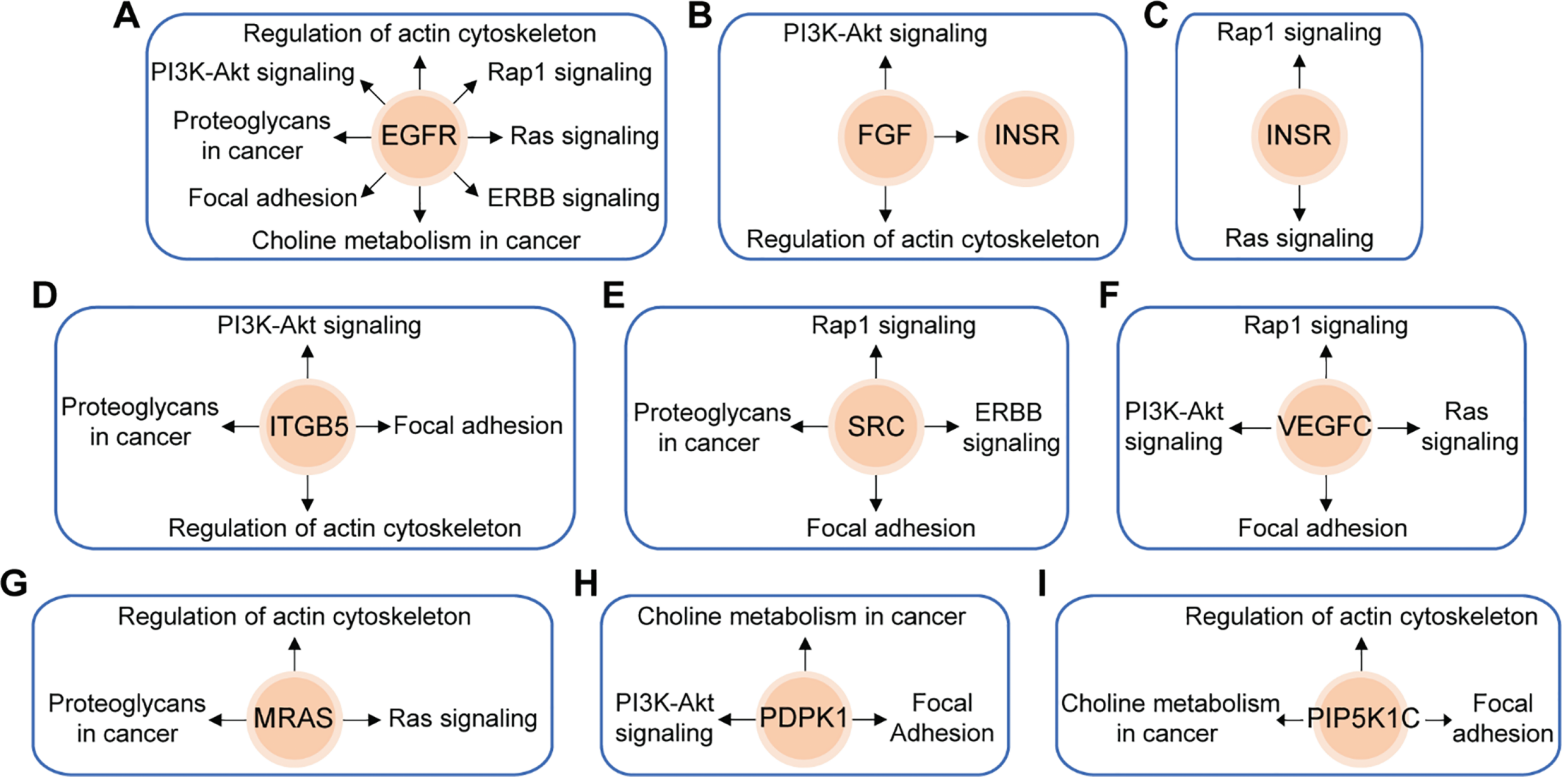
Schematic overviews of the nine hub genes and their associated KEGG signaling pathways. Nine target mRNAs of miR-99a-3p related to migration and invasion are closely linked to eight major downregulated signaling pathways significantly affected by HOXA10-AS knockdown. **(A)** EGFR is involved in all eight pathways. **(B)** FGF is implicated in two pathways and influences INSR. **(C)** INSR is associated with two pathways. **(D)** ITGB5 is connected to four pathways. **(E)** SRC is involved in four pathways. **(F)** VEGFC is linked to four pathways. **(G)** MRAS is associated with three pathways. **(H)** PDPK1 is connected to three pathways. **(I)** PIP5K1C is involved in three pathways. Orange circles represent the mRNAs, and black arrows indicate the signaling pathways in which these mRNAs were involved

**Figure 7:**
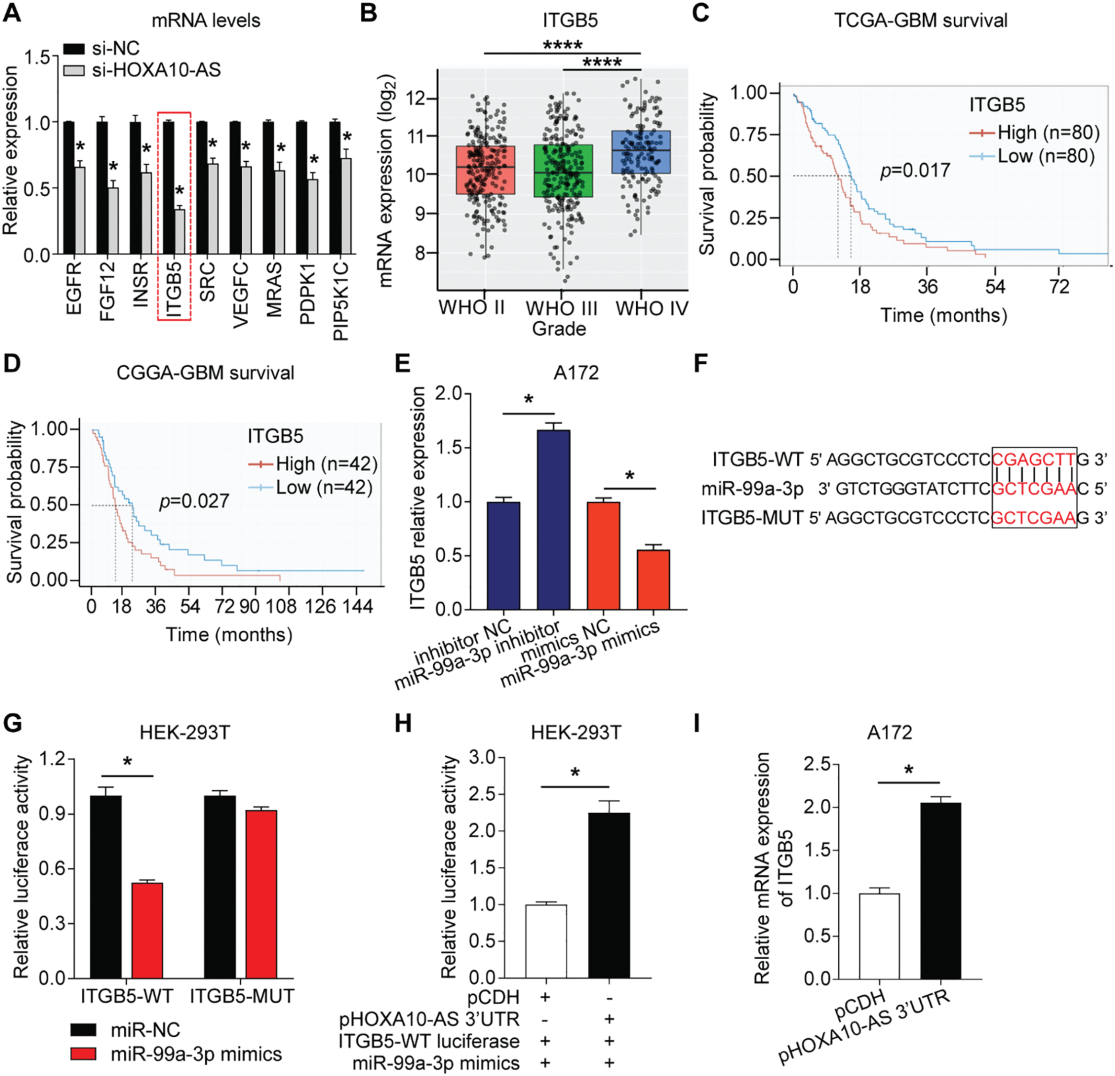
HOXA10-AS regulated ITGB5 by competitive binding to miR-99a-3p via the ceRNA mechanism. **(A)** Relative expression of 9 hub mRNAs in HOXA10-AS siRNA-transfected and control A172 cells. **(B)** ITBG5 expression in glioblastoma of different WHO grades from the TCGA database (Grade II: n = 226, Grade III: n = 244, Grade IV: n = 150). **(C-D)** Kaplan-Meier survival analysis based on ITGB5 expression in glioblastoma patients from the TCGA and CGGA datasets. **(E)** qRT-PCR was carried out to identify ITGB5 mRNA levels post-transfection with miR-99a-3p mimics or inhibitors. **(F)** ITGB5-WT/MUT luciferase plasmid was constructed for the miR-99a-3p binding site. **(G)** The binding between miR-99a-3p and ITGB5 was verified via dual-luciferase reporter assays. **(H)** Dual-luciferase reporter assays were conducted to verify if HOXA10-AS 3^′^UTR competitively binds miR-99a-3p. **(I)** qRT-PCR was carried out to elucidate ITGB5 levels in pHOXA10-AS 3^′^UTR transfected A172 cells. ****p < 0.0001, *p < 0.05

In this study, we utilized the Affymetrix Human Clariom D microarray to simultaneously analyze the changes in mRNA following the silencing of HOXA10-AS in A172 cells. Fig. 4A illustrates the schematic diagram of the workflow for data analysis. Compared to the control group, we identified 4556 differentially expressed mRNAs (|fold change| > 2, *p* < 0.05) after HOXA10-AS knockdown, of which 2468 consisted of downregulated mRNAs and 2088 upregulated mRNAs. Utilizing the miRanda software, we searched miR-99a-3p’s target genes among the 2468 significantly downregulated mRNAs and identified 188 mRNAs that are predicted to be miR-99a-3p’s target genes. Subsequently, KEGG pathway enrichment analysis revealed that among the 188 putative target mRNAs of miR-99a-3p, 37 mRNAs are implicated in tumor invasion and metastasis (Fig. 4B). Next, we conducted a KEGG pathway enrichment analysis on these 37 significantly downregulated mRNAs and revealed that 17 of them clustered within eight pathways related to cancer invasion and metastasis (Fig. 5A). These pathways include the regulation of focal adhesion, cytoskeleton, proteoglycans in cancer, choline metabolism in cancer, and Ras, PI3K-AKT, Rap1, and Erbb pathways. Within these eight pathways associated with tumor invasion and metastasis, each pathway contains at least four mRNAs whose expression significantly decreased due to HOXA10-AS knockdown, and these mRNAs are highlighted in yellow (Fig. 5B–D). Furthermore, among the 17 significantly downregulated mRNAs, nine were found to participate in more than two of the aforementioned cancer invasion and metastasis-related pathways, namely EGFR, FGF, INSR, ITGB5, SRC, VEGFC, MRAS, PDPK1, and PIP5K1C, which we refer to as hub genes (Fig. 6). Quantitative real-time PCR analysis revealed that these 9 hub genes exhibited significantly decreased expression in HOXA10-AS-knockdown A172 cells (Fig. 7A). In conclusion, these results suggest that these significantly downregulated target mRNAs predicted by miR-99a-3p, which are associated with tumor invasion and metastasis, may modulate HOXA10-AS-mediated metastasis and invasion.

### 3.5 HOXA10-AS Is an miR-99a-3p Sponge, Upregulating ITGB5 through a ceRNA Mechanism

Integrin subunit beta 5 (ITGB5) is a well-known gene associated with tumor invasion and metastasis [33,34]. It has been reported to exhibit oncogenic properties in various malignancies [33,34] and is linked to the prognosis of glioblastomas [35]. The study initially analyzed the TCGA database (grade II = 226 cases, grade III = 244 cases, grade IV = 150 cases) and found that ITGB5 levels were markedly higher in grade IV glioblastomas than in lower-grade glioblastomas (grades II and III) (Fig. 7B). This differential expression indicated that ITGB5 is crucially associated with tumor aggressiveness and the invasive characteristics of high-grade glioblastomas. Moreover, survival analysis from both the TCGA (n = 160) and CGGA (n = 84) datasets revealed that patients with high ITGB5 expression exhibited poor prognoses (Fig. 7C,D). The elevated levels of ITGB5 were associated with a more aggressive progression of the disease, underscoring its critical role in enhancing the invasiveness and metastatic potential of glioblastoma.

Next, the study assessed the impact of miR-99a-3p on ITGB5 expression. In A172 cells transfected with a miR-99a-3p inhibitor, the study observed an increase in ITGB5 expression, while miR-99a-3p mimics transfection reduced ITGB5 levels (Fig. 7E). These findings indicate that ITGB5 is regulated by miR-99a-3p. The dual-luciferase reporter test was carried out to validate whether miR-99a-3p directly binds ITGB5 3^′^UTR. An ITGB5 WT and MUT firefly luciferase plasmid was prepared with specificity for the binding site of miR-99a-3p within the ITGB5 3^′^UTR (Fig. 7F). HEK-293 T cells were co-transfected with the plasmids and either miR-NC or miR-99a-3p mimics. A significant reduction in luciferase activity was observed in the ITGB5-WT plasmid and miR-99a-3p mimic co-transfection group compared to controls, whereas the ITGB5-MUT and miR-99a-3p mimic co-transfected group’s luciferase activity showed no significant changes (Fig. 7G). These data indicate that ITGB5 is a target gene of miR-99a-3p.

To further evaluate whether HOXA10-AS 3^′^UTR exerts regulatory effects on ITGB5 expression via the ceRNA mechanism by competitively binding to miR-99a-3p, plasmids for overexpressing the HOXA10-AS 3^′^UTR (pHOXA10-AS 3^′^UTR) were constructed and cotransfected into HEK-293 T cells along with ITGB5-WT luciferase plasmid and miR-99a-3p mimics. Fig. 7H demonstrates a marked elevation in luciferase activity following co-transfection with pHOXA10-AS 3^′^UTR and ITGB5-WT, relative to control groups receiving pCDH and ITGB5-WT. These findings suggest that HOXA10-AS 3^′^UTR potentially functions as a competitive endogenous RNA for miR-99a-3p, attenuating miRNA-mediated suppression of ITGB5-WT expression and consequently enhancing reporter activity. Furthermore, upon upregulating the HOXA10-AS 3^′^UTR in A172 cells, ITGB5 transcript levels showed notable elevation vs. the control group (Fig. 7I). These findings revealed that HOXA10-AS can modulate ITGB5 expression via ceRNA-mediated competitive binding with miR-99a-3p.

### 3.6 Silencing HOXA10-AS Impaired Glioblastoma Cell Migration and Invasion, an Effect That Can Be Rescued by miR-99a-3p Inhibition or ITGB5 Overexpression

**Figure 8:**
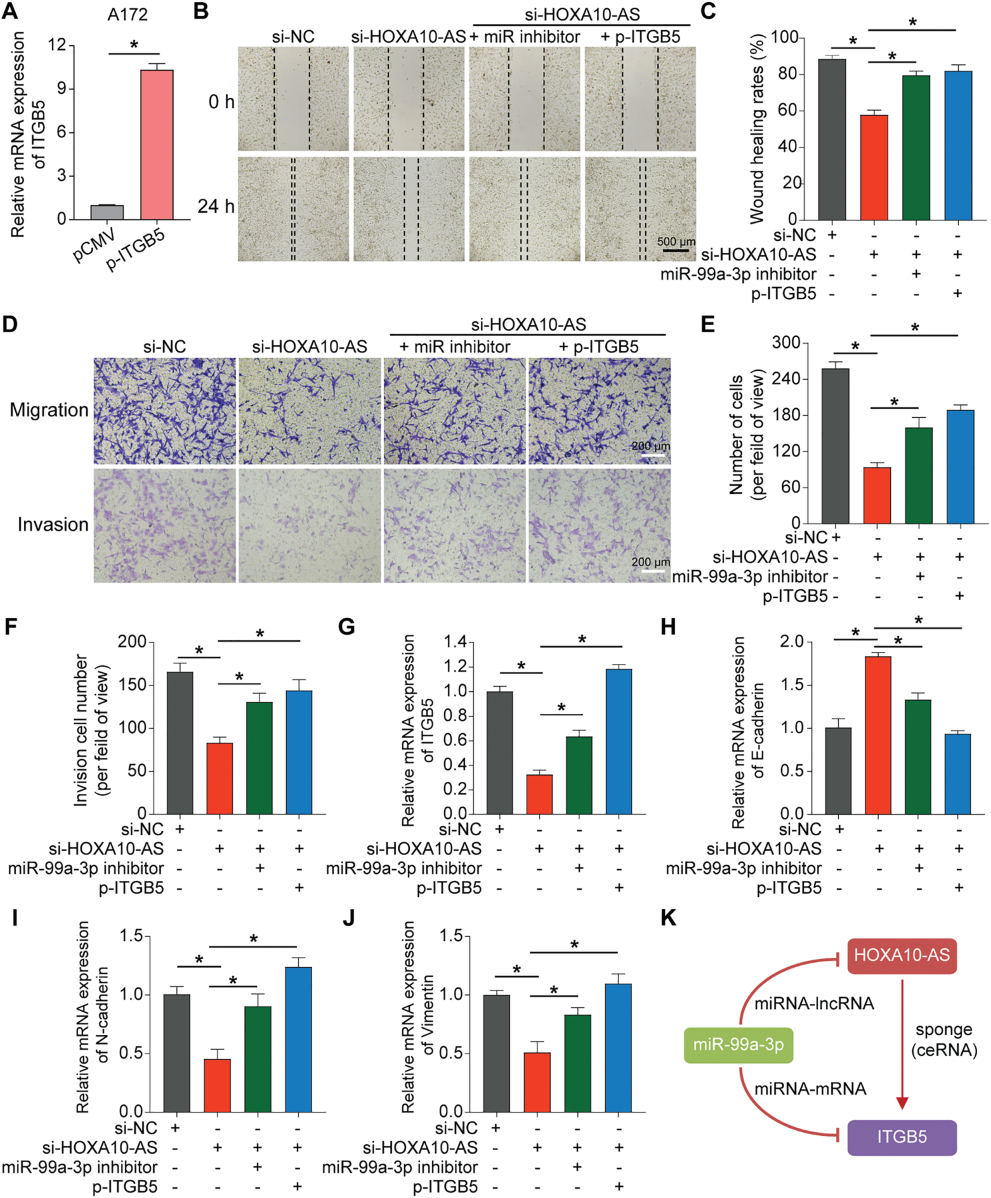
Overexpression of miR-99a-3p inhibitor or ITGB5 rescued the loss of function caused by HOXA10-AS knockdown. **(A)** ITGB5 expression was evaluated via qRT-PCR. **(B)** Wound healing analysis was carried out to evaluate migration ability. Scale bar: 500 μm. **(C)** Wound healing quantification results. **(D)** Transwell assays, both without and with Matrigel, were conducted to assess the migration and invasion capabilities. Scale bar: 200 μm. **(E)** Transwell migration quantification results. **(F)** Quantification of the Transwell invasion assays is shown. **(G)** qRT-PCR was performed to assess the expression level of ITGB5 in the four groups. **(H–J)** qRT-PCR was conducted to assess the expression levels of EMT-related markers E-cadherin, N-cadherin, and Vimentin in the four groups. **(K)** The lncRNA-miRNA-mRNA feed-forward loop of HOXA10-AS, miR99a-3p, and ITGB5. Values are indicated as mean ± SEM of 3 independent assays. *p < 0.05

To explore the regulatory effects of HOXA10-AS on ITGB5 expression and its functional consequences in glioblastoma, HOXA10-AS-knockdown cell lines were utilized for subsequent experiments; in parallel, an ITGB5-overexpressing plasmid was generated for mechanistic validation. After transfecting the ITGB5 overexpression plasmid into A172 cells, the mRNA levels of ITGB5 were significantly increased (Fig. 8A). Subsequently, four distinct experimental groups were created for A172 cells: control, HOXA10-AS knockdown, miR-99a-3p inhibitor-transfected HOXA10-AS knockdown, and ITGB5 overexpression plasmid-transfected HOXA10-AS knockdown group. Wound healing and Transwell assays were carried out to assess these four groups’ migratory and invasive abilities. As shown in Fig. 8B–F, the migratory and invasive capabilities of A172 cells were significantly diminished following HOXA10-AS knockdown compared to the control group. However, these capabilities were restored partially upon transfection with either the miR-99a-3p inhibitor or the ITGB5 overexpression plasmid. Concurrently, ITGB5 levels in all four groups were also assessed, which showed that ITGB5 expression was markedly reduced after HOXA10-AS knockdown. Conversely, transfection with the miR-99a-3p inhibitor or ITGB5 overexpression resulted in a recovery of ITGB5 expression levels (Fig. 8G). Additionally, we explored the effects of the miR-99a-3p inhibitor and ITGB5 overexpression on EMT by conducting rescue experiments with the aforementioned four groups. The qRT-PCR results demonstrated that both the miR-99a-3p inhibitor and ITGB5 overexpression significantly reversed the mRNA level changes induced by HOXA10-AS knockdown, specifically E-cadherin increment and the reduction of N-cadherin and Vimentin (Fig. 8H–J). These findings suggest that HOXA10-AS regulates ITGB5 levels by interacting competitively with miR-99a-3p, enhancing the migration and invasion of glioblastoma cells (Fig. 8K).

## 4 Discussion

Glioblastoma is a malignant brain tumor, and its capacity for invasion and migration is a major factor contributing to treatment failure and poor patient prognosis [36,37]. Understanding the mechanisms behind glioblastoma cell invasion and migration is crucial for developing new therapeutic strategies [38]. This study specifically examined the role and mechanisms of HOXA10-AS in glioblastoma cell invasion and migration. Our findings demonstrated that HOXA10-AS silencing markedly suppressed glioblastoma cells’ invasion, migration, and the associated EMT process. Furthermore, HOXA10-AS acts as a sponge for miR-99a-3p in glioblastoma cells through a ceRNA mechanism, leading to increased ITGB5 expression. Notably, the inhibition of migration and invasion due to HOXA10-AS knockdown could be rescued by a miR-99a-3p inhibitor or ITGB5 overexpression. These insights deepen our understanding of glioblastoma biology and suggest potential targets for therapeutic intervention.

Invasiveness is considered one of the most significant malignant traits of glioblastoma. In recent decades, lncRNAs have increasingly been recognized for their involvement in glioblastoma progression and metastasis [39,40]. This research investigation confirmed the critical involvement of HOXA10-AS in glioblastoma cells’ invasion and migration. This supports previous evidence of HOXA10-AS promoting tumor invasion in various cancers, including gastric cancer [41], lung adenocarcinoma [42], esophageal carcinoma [27], and nasopharyngeal cancer [43]. Furthermore, HOXA10-AS has been shown to enhance Wnt/β-catenin signaling in lung adenocarcinoma cell lines [42], consistent with our results demonstrating that HOXA10-AS downregulation significantly inhibits the EMT process. Our microarray analysis revealed that HOXA10-AS could regulate multiple hub genes and pathways associated with the migration and invasion of glioblastoma cells. As an lncRNA, HOXA10-AS likely promotes glioblastoma invasion by regulating key molecules in signaling pathways.

This study found that HOXA10-AS functions as a sponge for miR-99a-3p in glioblastoma cells, and we validated HOXA10-AS as a target gene of miR-99a-3p. This interaction indicated that miR-99a-3p may play a significant role in the pathogenesis and prognosis of glioblastoma. Previous studies have reported a potential link between miR-99a-3p and the pathogenesis of cancers. For example, miR-99a-3p regulates oncogenes in head and neck squamous cell carcinoma. Among its 32 target genes, 10 (including TIMP4, BCAT1, PDIA3, SUV420H1, HSP90B1, MTHFD2, STAMBP, CANX, TMEM14C, and SLC22A15) correlate with 5-year survival, likely by modulating cancer cell metastasis [44]. In addition to sponging lncRNA HOXA10-AS, miR-99a-3p has been found to bind lncRNA HOXC cluster antisense RNA 1 (HOXC-AS1), leading to the subsequent upregulation of matrix metalloproteinase 8 [45]. This underscores the multifaceted roles of miR-99a-3p in cancer progression and its critical interactions with lncRNAs in glioblastoma.

Increasing evidence suggests that HOXA10-AS expression is associated with poor survival outcomes in various malignancies [26,46]. Specifically, high levels of HOXA10-AS correlate with an unfavorable prognosis in both IDH-WT and IDH-MUT glioblastomas [26]. This study also found that ITGB5 is overexpressed in high-grade glioblastomas, and elevated ITGB5 expression is indicative of poor prognosis in both TCGA and CGGA patient databases. Additionally, ITGB5 has been shown to correlate with the mesenchymal subtype of glioblastoma and is involved in regulating immune responses and angiogenesis processes that are essential for glioblastoma cell migration and invasion [35]. Chen et al. have noted that upregulated ITGB5 expression is linked with highly enriched extracellular matrix receptor interaction pathways and focal adhesion, which are vital for tumor cells’ survival and invasion [47]. Furthermore, ITGB5 enhances the EMT process and the Wnt/β-catenin signaling pathway, thereby promoting tumor growth and metastasis [47]. Both HOXA10-AS and ITGB5 are highly expressed in glioblastoma, and their elevated expression is associated with unfavorable patient outcomes, providing a foundation for further investigation into their roles and interactions in this aggressive tumor type. Understanding the functional relationship between these molecules may uncover new therapeutic targets and strategies for improving glioblastoma treatment.

The ceRNA mechanism has been found to be widespread in tumor cells, underscoring its significance within the complex regulatory network [48]. HOXA10-AS has been reported to be located in the cytoplasm of leukemia cells [46]. A prior study has revealed that HOXA10-AS may act as an oncogene by binding to miR-6509-5p, leading to the upregulation of Y-box binding protein 1 in gastric cancer [41]. Through luciferase assays and rescue functional experiments, we identified ITGB5 as a target gene of miR-99a-3p. Additionally, we demonstrated that HOXA10-AS can regulate ITGB5 expression by competitive binding to miR-99a-3p through a ceRNA mechanism. This finding provides new insights into the role of HOXA10-AS in mediating the invasion and migration of glioblastoma cells. The interactions among lncRNAs, miRNAs, and mRNAs highlight a critical layer of post-transcriptional regulation that may contribute to glioblastoma cell invasion and migration.

However, this study has several limitations. First, while we demonstrated that HOXA10-AS knockdown leads to decreased invasion and migration of glioblastoma cells through ITGB5-related pathways, additional pathways warrant further exploration. Second, to clarify the detailed mechanisms by which ITGB5 facilitates the pathological process of glioblastoma, more extensive research is needed. Third, the Kaplan-Meier analyses regarding ITGB5 were conducted using data derived from databases. Incorporating multivariate analysis to control for confounding factors, such as glioblastoma molecular subtypes or therapeutic interventions, could strengthen the findings. Additionally, further *in vivo* validation and studies in other cell lines are necessary to enhance the translational applicability of our discoveries. To address these limitations and bridge current knowledge gaps, future studies should focus on elucidating the broader regulatory network of HOXA10-AS, including its potential interactions with additional hub genes, miRNAs, or signaling pathways, such as Wnt/β-catenin or PI3K-AKT, to comprehensively define its role in glioblastoma progression. Furthermore, preclinical evaluations of therapeutic strategies targeting the HOXA10-AS/miR-99a-3p/ITGB5 axis, including antisense oligonucleotides or nanoparticle-based miRNA delivery systems, are warranted to assess their translational potential in improving glioblastoma treatment outcomes.

## 5 Conclusions

Knocking down HOXA10-AS substantially suppressed glioblastoma cells’ invasion and migration. We also investigated the molecular mechanism involved and demonstrated that HOXA10-AS might function as a ceRNA by sponging miR-99a-3p, thereby reducing its inhibitory effect on ITGB5. Our study reveals a novel regulatory network in glioblastoma cells that enhances our understanding of the pathogenesis of glioblastoma and identifies HOXA10-AS as a potential new therapeutic target for glioblastoma treatment.

## Data Availability

The datasets generated and/or analyzed during this study are available from the corresponding author upon reasonable request.
